# Comment on “Inverse relationship between serum haptoglobin and acute kidney injury in critically III patients with sepsis: A retrospective cohort study of the MIMIC-IV 3.0 database”

**DOI:** 10.1016/j.clinsp.2026.100951

**Published:** 2026-04-14

**Authors:** Preeti Lamba

**Affiliations:** Dr D.Y. Patil Vidyapeeth University, Dr D.Y. Patil Medical College Hospital and Research Centre, Pune, Maharashtra, India

*Dear Editor,*

I endorse Liao et al. for their pertinent study of serum haptoglobin in relation to Acute Kidney Injury (AKI) in severely ill septic patients, using freely available MIMIC-IV 3.0 database. AKI is one of the most life-threatening complications of sepsis. It can improve the outcome if it early identifies the high-risk patients. The above study done in the intensive care unit had showed that how this affordable and widely available biomarker may help to improve the risk assessment.

The authors showed that a greater rate of AKI was independently correlated with lower haptoglobin levels. Given the vital function of haptoglobin in binding free hemoglobin, reducing oxidative kidney damage and lowering tubular tension, this inverse correlation is biologically sensible. Their result’s statistical reliability is improved by the inclusion of several covariates helps reduce confounding and the use of a big retrospective cohort [1, 2, 3, 4]

But some very important things come to mind.

First, haptoglobin being an acute-phase reactant; systemic inflammation, liver function, hemolysis or chronic illnesses may affect its plasma levels. These are all things are commonly seen in septic patients. The authors left out some conditions, but more stratified studies could make these interactions clearer.

Second, relying on only one laboratory test at ICU admission reduces understanding of dynamic changes as variations in haptoglobin across the septic course may give extra predictive value.

Third, comparing haptoglobin with other AKI indicators like Neutrophil Gelatinase-Associated Lipocalin (NGAL), cystatin C or Kidney Injury Molecule-1 (KIM-1) would assist to find out whether it provides little prognostic value.

Finally, Liao et al. offer some early evidence suggesting that in septic patients’ serum haptoglobin may be a useful supplementary indicator for early AKI identification. Further future studies and external validation could enable haptoglobin to be included in clinical decision-making channels to enhance results in crucial care nephrology.

## Ethical approval

Not applicable.

## Funding

No funding was received for this research.

## Data availability

The datasets generated and/or analyzed during the current study are available from the corresponding author upon reasonable request.

## Declaration of competing interest

The authors declare no conflicts of interest.
